# *IGF-1* Genome-Edited Human MSCs Exhibit Robust Anti-Arthritogenicity in Collagen-Induced Arthritis

**DOI:** 10.3390/ijms25084442

**Published:** 2024-04-18

**Authors:** Dong-Sik Chae, Seongho Han, Sung-Whan Kim

**Affiliations:** 1 Department of Orthopedic Surgery, Catholic Kwandong University College of Medicine, International St. Mary’s Hospital, Incheon 22711, Republic of Korea; 2 Department of Family Medicine, Dong-A University College of Medicine, Dong-A University Medical Center, Busan 49236, Republic of Korea; 3 Department Medicine, Catholic Kwandong University College of Medicine, Gangneung 25601, Republic of Korea

**Keywords:** arthritis, cell therapy, genome editing, IGF-1, stem cells

## Abstract

Stem cell therapy stands out as a promising avenue for addressing arthritis treatment. However, its therapeutic efficacy requires further enhancement. In this study, we investigated the anti-arthritogenic potential of human amniotic mesenchymal stem cells (AMM) overexpressing insulin-like growth factor 1 (IGF-1) in a collagen-induced mouse model. The *IGF-1* gene was introduced into the genome of AMM through transcription activator-like effector nucleases (TALENs). We assessed the in vitro immunomodulatory properties and in vivo anti-arthritogenic effects of IGF-1-overexpressing AMM (AMM/I). Co-culture of AMM/I with interleukin (IL)-1β-treated synovial fibroblasts significantly suppressed NF-kB levels. Transplantation of AMM/I into mice with collagen-induced arthritis (CIA) led to significant attenuation of CIA progression. Furthermore, AMM/I administration resulted in the expansion of regulatory T-cell populations and suppression of T-helper-17 cell activation in CIA mice. In addition, AMM/I transplantation led to an increase in proteoglycan expression within cartilage and reduced infiltration by inflammatory cells and also levels of pro-inflammatory factors including cyclooxygenase-2 (COX-2), IL-1β, NF-kB, and tumor necrosis factor (TNF)-α. In conclusion, our findings suggest that *IGF-1* gene-edited human AMM represent a novel alternative therapeutic strategy for the treatment of arthritis.

## 1. Introduction

Rheumatoid arthritis (RA) is a debilitating chronic condition characterized by joint lesions and progressive destruction of articular cartilage, ultimately resulting in the impairment of joint function [1]. The management of RA poses significant challenges to healthcare systems because of its chronic nature and associated complications. Current therapeutic strategies primarily involve the use of anti-rheumatic drugs such as blockade of interleukin (IL)-1 and IL-6 or tumor necrosis factor (TNF)-α inhibitors [2]. However, these interventions are fraught with issues such as high costs, adverse effects, and prolonged treatment duration. Hence, there is an urgent imperative to explore novel therapeutic modalities with enhanced efficacy and safety profiles for the management of RA patients.

RA progression is closely associated with dysregulation of pro-inflammatory cytokines, with aberrant activation of macrophages or T-cells contributing to their production [3]. Additionally, IL-17 has been implicated in triggering pro-inflammatory responses in synovial fibroblasts [4]. Stem cell therapy, particularly the use of mesenchymal stem cells (MSCs), has emerged as a promising approach for the treatment of RA. MSCs demonstrate the capacity to modulate immune responses through the release of anti-inflammatory factors or by indirectly facilitating the polarization of regulatory T-cells (Tregs) or macrophages [5]. Notably, MSC-based therapy has been utilized for knee RA management, demonstrating various therapeutic benefits such as pain alleviation, mild immune modulation, partial restoration of damaged cartilage, and improved joint function, with minimal adverse effects [6,7]. Nevertheless, further studies employing cutting-edge technologies are warranted to enhance the efficacy and safety profile of MSC-based therapies for RA patients.

Insulin-like growth factor (IGF)-1, which belongs to a family of growth factors structurally akin to pro-insulin, exerts significant effects on the biological behavior of chondrocytes [8]. It plays a fundamental role in regulating cartilage matrix metabolism, particularly during cartilage repair. IGF-1 exerts potent effects by significantly enhancing synthesis of the cartilage matrix, while concurrently inhibiting its degradation. In vitro studies have demonstrated that IGF-1 plays a pivotal role in promoting the synthesis of cartilage and proteoglycan [9]. IGF-1 also mitigates inflammation induced by oxidized low-density lipoproteins by reducing the release of high-mobility group box 1 protein [10]. IGF-1-overexpressing constructs demonstrate significant enhancement in promoting osteochondral repair while concurrently reducing cartilage degeneration proximal to the defect site [8].

Our recent work has led to the development of a robust gene expression system utilizing transcription activator-like effector nucleases (TALENs) to establish stable cytokine secretion after genomic insertion of *IGF-1* into human amniotic mesenchymal stem cells (AMM) [11,12]. Building on this advancement, we explored the anti-arthritogenic potential of *IGF-1* gene-edited amniotic MSCs (AMM/I) in an experimental arthritis model with cartilage damage.

## 2. Results

### 2.1. Targeted Knock-In of IGF-1

**Figure 1:**
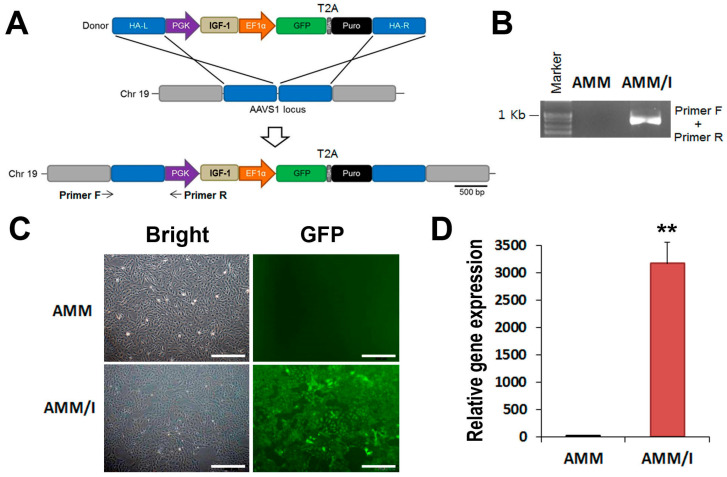
Generation of AMM/I cell line using TALEN gene editing. (**A**) Schematic picture of the donor vector carrying IGF-1 donor plasmid DNA. The expression cassette containing the PGK promoter-driven IGF-1 and EF1α promoter-driven GFP-T2A-puromycin was inserted into the AAVS1 site via homology-directed repair (HR). The locations of primers for junction detection are indicated (primers F and R). Abbreviations: HA-L, left homology arm; HA-R, right homology arm; PGK, phosphoglycerate kinase promoter; EF1α, elongation factor-1 alpha promoter; Puro, puromycin. (**B**) Inserted donor plasmid was confirmed in control AMM and AMM/I using junction PCR. (**C**) GFP expressing AMM/I. Transfected cells were selected based on puromycin, followed by FACS sorting. Bars = 500 μm. (**D**) Expression levels of IGF-1 were examined using q-PCR. ** *p* < 0.01, *n* = 4.

In this study, we used TALENs technology to introduce *IGF-1* into human amniotic mesenchymal stem cells (AMM) in a targeted manner. The donor plasmid used to target *IGF-1* was driven by the phosphoglycerate kinase promoter, along with elongation factor-1-alpha and green fluorescent protein (GFP)–T2A–puromycin (Figure 1A). Following transfection of AMM with both TALENs and donor plasmids, the cells were subjected to puromycin selection, resulting in a survival rate of 44.3%. Subsequently, fluorescence-activated cell sorting was employed to isolate 99.1% of GFP-positive cells (Figure 1B). The accuracy of donor plasmid insertion was validated through amplification of the junction fragment (960 bp), confirming correct integration (Figure 1C). Furthermore, quantitative reverse transcription-polymerase chain reaction analysis demonstrated a significant elevation in *IGF-1* levels in *IGF-1* knock-in AMM (AMM/I) compared to their non-transfected counterparts (Figure 1D). AMM/I was used in this study.

### 2.2. Characteristics of AMM/I

Cultured AMM/I exhibited a spindle fibroblast-like morphology similar to that of AMM (Supplemental Figure S1A). Flow cytometric analysis revealed that AMM/I retained the characteristic expression profile of mesenchymal stem cells (MSCs), with high expression levels of MSC markers (CD29, CD73, and CD90) and minimal expression of hematopoietic cell markers (CD14 and CD45) (Supplemental Figure S1B).

### 2.3. AMM/I Immunomodulatory Properties

**Figure 2:**
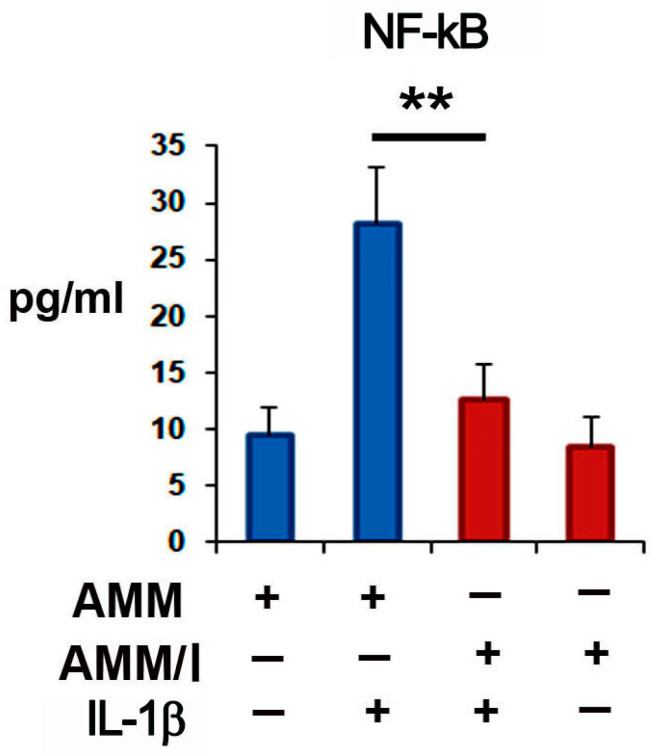
Immunomodulatory potential of IGF-1-overexpressing human amniotic mesenchymal stem cells (AMM/I) in vitro. AMM and AMM/I were treated or not with IL-1β for 1 day and were then co-cultured with synovial fibroblasts for 2 days. The supernatants were collected and the concentrations of NF-kB were measured via enzyme-linked immunosorbent assay (ELISA) (*n* = 5 each; ** *p* < 0.01). Co-culture with AMM/I and synovial fibroblasts significantly decreased IL-1β levels compared to co-culture with AMM.

To assess the immunomodulatory potentials of AMM/I on T-cells in vitro, AMM/I and AMM were treated with or without IL-1β and co-cultured with synovial fibroblasts. Cytokine levels in the supernatants of these co-cultures were measured after two days. Enzyme-linked immunosorbent assays demonstrated that co-culture with AMM/I significantly decreased NF-kB levels compared to co-culture with AMM alone (Figure 2).

### 2.4. In Vivo AMM/I Anti-Arthritogenic Properties

**Figure 3:**
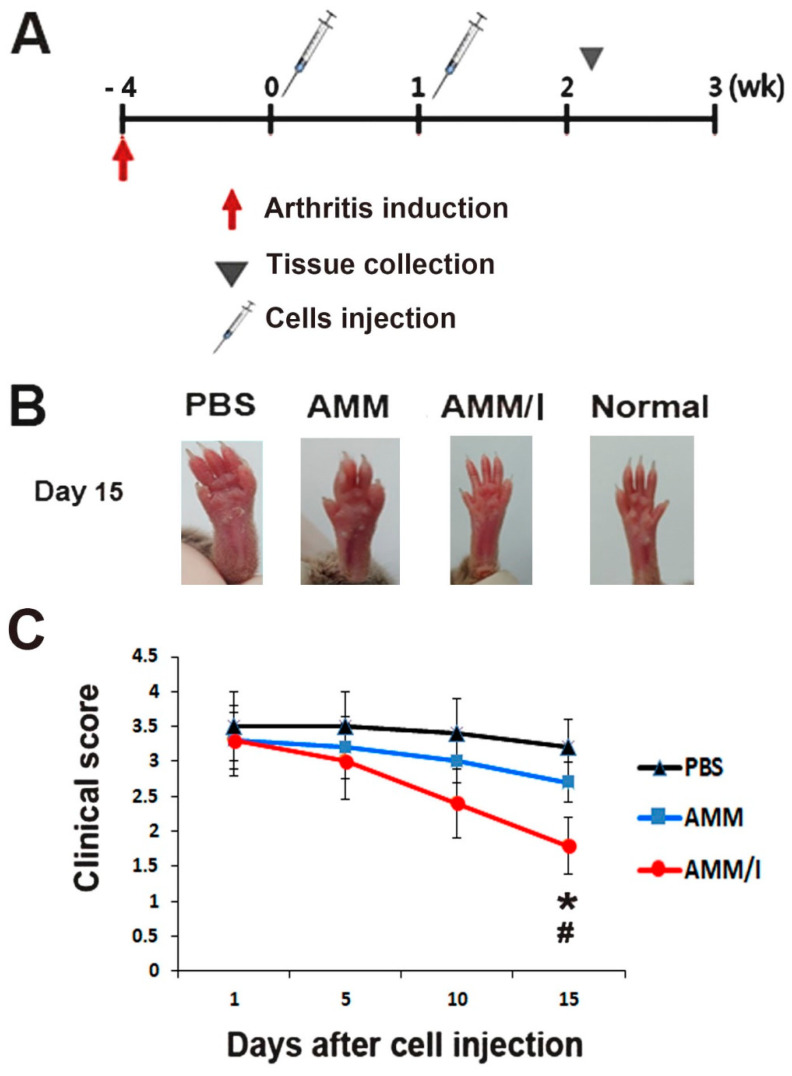
AMM/I transplantation effectively prevents disease progression. (**A**) The schematic illustrates the procedures for arthritis induction, cell injection, and specimen collection. (**B**) Representative images depict the paws of mice post-cell injection. (**C**) Arthritis scores were quantified based on the severity of paw swelling (# *p* < 0.01 AMM/I vs. PBS, * *p* < 0.05, AMM/I vs. AMM, *n* = 5). The arthritis clinical score exhibited a significant reduction 15 days after AMM/I injection compared to both PBS-injected and AMM-treated groups.

To assess the anti-arthritogenic potential of AMM/I in vivo, we used a collagen-induced arthritis (CIA) mouse model by inducing arthritis in mouse paws. Subsequently, the mice were intraperitoneally administered AMM/I, AMM, or phosphate-buffered saline (PBS) twice weekly (Figure 3A). Joint tissues were collected 15 days post-injection. Remarkably, arthritis clinical scores exhibited a significant reduction 15 days after AMM/I injection compared to both the PBS-injected and AMM-treated groups (Figure 3B,C).

**Figure 4:**
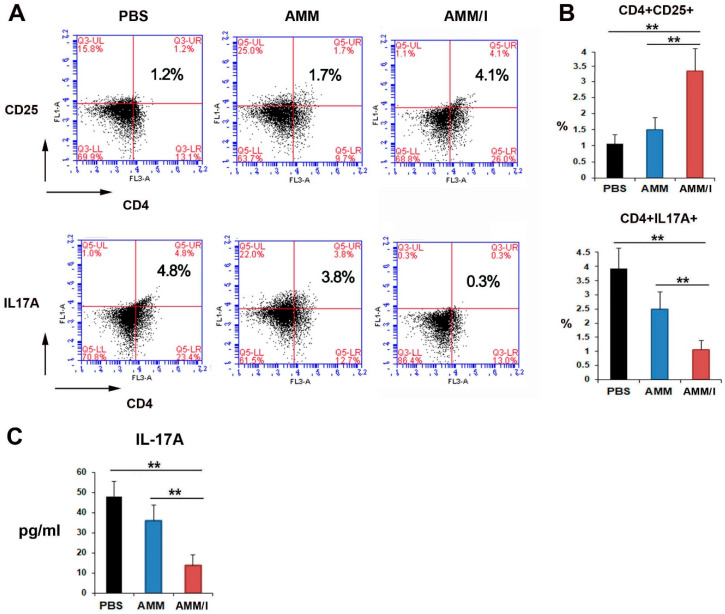
AMM/I transplantation modulates Tregs and Th-17 cell populations in collagen-induced arthritis (CIA) mice. (**A**) Flow cytometry data illustrating the identification of Treg and Th-17 cells derived from blood samples taken from CIA mice 14 days after cell or PBS injection. (**B**) Quantitative flow cytometry analysis of Tregs and Th-17 cells was conducted (*n* = 5 each; ** *p* < 0.01). AMM/I injection significantly reduced IL-17A levels compared to AMM or PBS injection. (**C**) Serum IL-17A concentration. Two weeks after cell injection, ELISA was conducted using the serum of mice. ** *p* < 0.01, *n* = 6 each.

Further investigation into the mechanisms underlying the anti-arthritogenic effects of AMM/I, we analyzed T-cell populations in blood. Flow cytometry analysis showed that injection of AMM/I led to an increase in the population of regulatory Tregs compared to PBS or AMM injections (Figure 4A). Conversely, the population of T helper 17 (Th-17) cells was notably decreased in the AMM/I-treated group (Figure 4B). Additionally, the level of IL-17A in the serum of the CIA mice was measured after cell injection. Notably, AMM/I injection significantly reduced IL-17A levels compared with PBS or AMM injections (Figure 3C).

### 2.5. Histological Analysis of Joints

**Figure 5:**
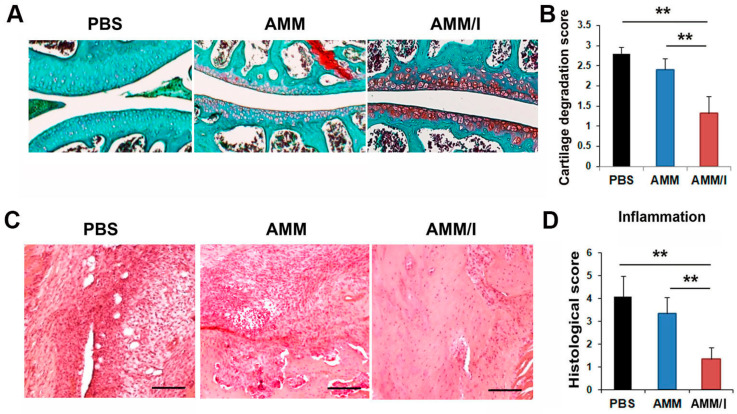
Histological examination of CIA mouse joints following cell injection. (**A**) Safranin O/fast green staining reveals proteoglycan expression in CIA mouse joints post-cell injection. Increased proteoglycan expression was observed in the articular cartilage of the AMM/I-treated group compared to the AMM- and PBS-treated control groups. Scale bars = 200 μm. (**B**) Quantitative assessment of cartilage degradation scores, with loss of proteoglycans indicated by staining intensity (*n* = 5 each; ** *p* < 0.01). (**C**) Representative images of joint tissue sections stained with hematoxylin and eosin (H&E). Histological analysis revealed that articular tissues injected with AMM/I exhibited significantly reduced inflammatory cell infiltration compared to those treated with AMM or PBS. Scale bars = 200 μm. (**D**) Quantification of inflammatory pathological scores, assessing mononuclear cell infiltration and tissue inflammation post-cell transplantation. AMM/I-injected tissues exhibited reduced mononuclear cell infiltration and maintained normal cartilage surface morphology (*n* = 5 each; ** *p* < 0.01).

To assess potential cartilage protection in vivo, a histological examination of joint tissues from CIA mice was conducted. Staining with Safranin O/fast green revealed increased proteoglycan expression within the articular cartilage of the AMM/I-treated group compared to that in both the AMM- and PBS-treated control groups, indicating the protective effects of AMM/I against cartilage damage (Figure 5A,B).

Hematoxylin and eosin staining was conducted to evaluate inflammatory responses after cell injection. Histological analysis demonstrated that articular tissues injected with AMM/I exhibited significantly reduced inflammatory cell infiltration compared to those treated with PBS or AMM (Figure 5C,D).

### 2.6. Anti-Arthritogenic Therapeutic Mechanism

**Figure 6:**
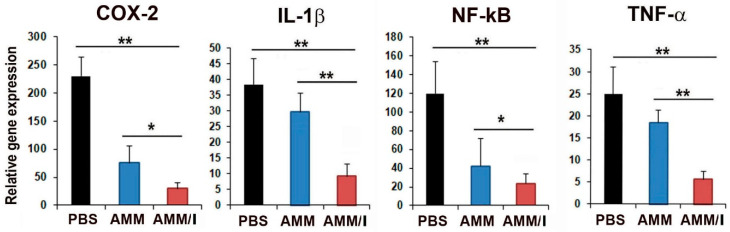
AMM/I transplantation mitigates inflammation in mouse joints. Quantitative reverse transcription-polymerase chain reaction (qRT-PCR) analyses were conducted on joint tissues injected with phosphate-buffered saline (PBS), AMM, and AMM/I. Transplantation of AMM/I led to reduced expression levels of key pro-inflammatory factors, indicating suppression of inflammation (*n* = 5 each; * *p* < 0.05, ** *p* < 0.01).

To further explore the therapeutic mechanisms involving AMM/I, we conducted gene expression analysis of pro-inflammatory factors in articular tissues from CIA mice four weeks after cell injection. RT-qPCR results showed that expression levels of pro-inflammatory factors including *COX-2*, *IL-1β*, *NF-kB*, and *TNF-α* were significantly diminished in AMM/I-injected articular tissues (Figure 6).

## 3. Discussion

In recent years, mesenchymal stem cells (MSCs) come to be regarded as promising candidates for cartilage regeneration and protection. However, their therapeutic efficacy requires further augmentation. Our study suggests that enhancing the expression of IGF-1 could potentiate the therapeutic capabilities of MSCs in addressing cartilage damage. We examined the anti-arthritogenic properties of *IGF-1*-modified amniotic mesenchymal stem cells (AMM/I) in an experimental arthritis model.

The therapeutic property of MSCs in rheumatic diseases has garnered attention, with notable studies demonstrating their efficacy in knee RA treatment [13]. In fact, transplantation of human MSCs reduces the levels of inflammatory factors such as IL-6, MCP-1, and TNF-α in CIA mice [14]. However, conflicting reports have suggested limited or negligible therapeutic effects in CIA mice [15,16,17]. It has been proposed that the immunosuppressive attributes of MSCs may not suffice to fully counteract arthritic conditions, particularly in the presence of elevated levels of IL-6 and TNF-α [17]. Consequently, there is an urgent need to bolster the therapeutic capacity of MSCs in the treatment of arthritis. Our objective was to develop safe and efficient strategies for enhancing the therapeutic properties of MSCs. Although AMM have shown promise in mitigating RA progression, their efficacy in cartilage repair remains limited because of their inherently low therapeutic potential. Thus, we endeavored to engineer more safer and potent stem cell lines through gene editing with a specific focus on enhancing cartilage protection and repair capabilities.

To augment the therapeutic capacity of stem cells while mitigating the risk of aberrant mutations, we devised a gene knock-in system employing AMM and a precise gene-editing technique [11]. AMM have been extensively investigated for their potential use in allogeneic stem cell therapy because of their immune-privileged nature and remarkable differentiation capabilities [18]. Moreover, AMM exhibit high gene insertion efficiency and robust cellular proliferation, rendering them particularly suitable candidates for gene editing applications. Hence, we opted to use AMM as the foundation for our research in this study.

IGF-1, belong to the growth factor family, exerts a significant influence on chondrocyte behavior and plays a pivotal role in regulating cartilage matrix metabolism during the repair process [8]. It induces chondrogenic differentiation and stimulates type II collagen synthesis. Effective strategies for delivering IGF-1 to chondrocytes and the cartilage matrix are crucial for its clinical application in the treatment of arthritis. In a study utilizing MSCs infected with an IGF-1 adenoviral vector and injected into damaged rat cartilage, significant production of type II collagen and satisfactory repair outcomes were observed [19]. The role of IGF-1 in mitigating IL-1-induced matrix degradation is well-established [20]. Moreover, studies have demonstrated its ability to attenuate IL-1β-induced activation of NF-κB by inhibiting IκB-α kinase. Our research further corroborates these findings, revealing that co-culture with splenocytes and AMM/I significantly suppressed NF-κB expression in an IL-1β-induced inflammatory milieu. Given the pivotal role of NF-κB-regulated gene products in cartilage degradation, inflammation, and apoptosis [21], inhibition of NF-κB shows promise in treating conditions such as RA or osteoarthritis (OA). Numerous NF-κB inhibitors have been reported to be in association with RA or OA [21], suggesting the potential utility of AMM/I as a pharmacological agent for NF-κB inhibition.

T-cells play crucial roles in regulating the inflammatory responses observed in RA. Specifically, Tregs play pivotal roles in preventing autoimmunity [22]. In addition, research has shown that IL-17-producing Th-17 cells are implicated in autoimmune conditions and in the pathogenesis of CIA [23]. IL-17 stimulates synoviocytes to produce inflammatory cytokines. IGF-1 has been shown to prevent T-cells [24] and downregulate pro-inflammatory cytokine signaling in articular cartilage [25]. IGF-1 also regulates Th-17 and Tregs [26]. In addition, IGF-1R signaling contributes to T-cell-dependent inflammation in RA [27]. Our findings align with those of previous research, as we observed a downregulation of Th-17 cells and inflammatory factors following the administration of AMM/I, suggesting the potential suppression of arthritis through the action of IGF-1 derived from AMM/I.

Recent studies have shown that administration of IGF-1 leads to an increase in the number of circulating CD4+ T-cells, with IGF playing a role in the induction of Tregs [27,28]. Additionally, induction of Treg cells was augmented by the addition of IGF and hindered by the inhibition of IGF-1R, suggesting a regulatory role for IGF-1 in this process [28]. In accord with these reports, we also observed the induction of Tregs in CIA mice treated with IGF-1-secreting AMM/I. These results indicate that the anti-inflammatory effects of IGF-1-expressing AMM/I may involve modulation of the Th-17/Treg cell balance in CIA.

Proteoglycans are indispensable constituents of the extracellular matrix (ECM) and are fundamental for maintaining tissue integrity and functionality. A noteworthy ex vivo investigation revealed that IGF-1, found in fetal bovine serum [29], plays a crucial role in sustaining proteoglycan synthesis in articular cartilage. Consequently, IGF-1 has emerged as a promising candidate for facilitating the regeneration of articular cartilage after injury [30]. Moreover, studies have demonstrated that both synovial fluid and serum from patients with RA fail to induce chondrocyte proteoglycan synthesis by blocking the function of IGF-1 using a primary antibody [31], underscoring the pivotal role of IGF-1 in cartilage matrix anabolism. This multifaceted function highlights the potential of IGF-1 as a therapeutic target to augment cartilage repair and regeneration. In agreement with these findings, transplantation of AMM/I resulted in a significant increase in proteoglycan expression and a reduction in CIA progression compared to AMM alone in articular cartilage. This highlights the augmented anti-arthritic efficacy of the AMM/I treatment strategy, suggesting that IGF-1 plays a pivotal role in protecting against cartilage degradation by bolstering chondrocyte viability and facilitating the synthesis of cartilage matrix components.

High levels of pro-inflammatory factors have been detected in the synovial fluid of patients with RA [32,33]. Decreased levels of IGF-1 have been found in patients with RA, suggesting an association with RA pathogenesis [34]. IGF-1 plays a pivotal role in conferring chondroprotective effects in inflammatory arthritis [35]. IGF-1 also reduces synovial inflammation in an equine model [36]. Transplantation of IGF-1-treated MSCs has been shown to suppress the production of inflammatory factors such as IL-1β, IL-6 and TNF-α at both protein and gene expression levels [37]. Consistent with these findings, we also observed reduced levels of the inflammatory factor NF-kB in co-cultures of AMM/I cells and splenocytes under in vitro inflammatory conditions. AMM/I transplantation also highly downregulated levels of the proinflammatory factors COX-2, IL-1β, NF-kB, and TNF-α in contrast to AMM in CIA mice. These results suggested that sustained exposure to IGF-1 augmented the anti-arthritogenic potential of AMM.

## 4. Materials and Methods

### 4.1. Cell Culture

Human amniotic mesenchymal stem cells (AMM) were procured from Thermo Scientific, Inc. (Waltham, MA, USA) and maintained in low-glucose Dulbecco’s Modified Eagle Medium (DMEM; GIBCO, Grand Island, NY, USA) supplemented with 100 U/mL penicillin, 100 µg/mL streptomycin (GIBCO), and 10% fetal bovine serum (FBS).

### 4.2. Construction of Donor Vector, Transfection, and Selection

The AMM/I cell line was generated following previously outlined methods [15]. In brief, IGF-1 was synthesized and integrated into adeno-associated virus integration site 1 (AAVS1) within the donor vector (System Biosciences, Palo Alto, CA, USA), targeting the NdeI and SalI restriction sites (Figure 1A). Following the construction of the donor vector, AMM (1 × 10^5^) were suspended in 10 μL electroporation buffer along with 0.6 μg of AAVS1 HR Donor, AAVS1 left, and right TALE-Nuclease vector (System Biosciences). Electroporation was performed using the Neon Transfection System (Thermo Fisher Scientific, Waltham, MA, USA), followed by selection of IGF-1 knock-in AMM by incubation with 5 μg/mL puromycin for 10 days, as described previously. Puromycin-selected cells were resuspended in fluorescence-activated cell sorting (FACS) buffer and sorted by FACS.

### 4.3. Junction PCR

Genomic DNA was extracted from AMM or AMM/I using the G-spin™ Total DNA Extraction Mini Kit (Intron Biotechnology, Suwon, Republic of Korea) as previously described [38]. Subsequently, a touch-down PCR approach was employed to amplify 120 ng of genomic DNA, involving a total of 36 cycles. The touch-down PCR protocol consisted of an initial cycle at 98 °C for 30 s, followed by 22 cycles comprising 98 °C for 30 s, 72–60 °C for 30 s (with a 1 °C decrease every two cycles), and 72 °C for 1 min. This was succeeded by 14 additional cycles at 98 °C for 30 s, 60 °C for 30 s, and 72 °C for 1 min, with a final step at 72 °C for 10 min. For the second PCR, 0.5 μL of the touch-down PCR product was utilized. The second PCR conditions consisted of an initial cycle at 98 °C for 30 s, followed by 35 cycles at 98 °C for 30 s, 65 °C for 30 s, and 72 °C for 1 min, with a final step at 72 °C for 10 min [38].

### 4.4. Quantitative Reverse Transcription PCR (RT-qPCR)

RT-qPCR assays were conducted according to previously established protocols [16,17]. Initially, total RNA was extracted from the cells using an RNA-stat (Iso-Tex Diagnostics, Friendswood, TX, USA), and the isolated RNA was reverse-transcribed using TaqMan reagent (Applied Biosystems, Foster City, CA, USA). The resulting cDNA was subjected to qRT-PCR using specific primers and probes. RNA levels were quantified using an ABI PRISM 7000 instrument (Applied Biosystems) and normalization to GAPDH expression. The qRT-PCR primers utilized were as follows: human IGF-1 (Hs00961622_m1), GAPDH (Hs99999905_m1), mouse IL-1β (Mm00434228_m1), COX2 (Mm03294838_g1), NF-kB (Mm00476361_m1), TNF-α (Mm00443258_m1), and GAPDH (Mm99999915_g1), all procured from Applied Biosystems.

### 4.5. Isolation of Synovial Fibroblasts

Synovial fibroblasts were obtained from the synovial tissue of DBA/1 mice (OrientBio, Seongnam, Republic of Korea) using a previously described method [39]. Briefly, synovial tissue was minced and filtered through a sterile 100 μm nylon filter (BD Biosciences, San Jose, CA, USA). These cells were cultured using Dulbecco’s modified Eagle’s medium. Prior to experimental use, cells were passaged several times. These cells exhibited a typical synovial fibroblast morphology and we confirmed the expression of vascular cell adhesion molecule-1 by immunohistochemical analysis.

### 4.6. Co-Culture and Enzyme-Linked Immunosorbent Assay (ELISA)

To assess the impact of AMM/I on synovial fibroblasts, 1 × 10^6^ AMM or AMM/I were treated with or without 10 ng/mL IL-1β for one day, followed by co-culture with 1 × 10^6^ splenocytes in RPMI 1640 supplemented with 10% FBS. Supernatants from the co-cultures were collected after 2 days and cytokine levels were measured. Cytokine levels in the supernatants were assessed using a murine NF-kB ELISA kit (Thermo Fisher Scientific).

### 4.7. Induction of Collagen-Induced Arthritis Model and Treatment

Bovine type II collagen (Chondrex, Redmond, WA, USA) was emulsified in a 1:1 ratio with complete Freund’s adjuvant (Chondrex) containing 2 mg/mL of heat-killed Mycobacterium tuberculosis. Six-week-old male DBA/1 mice (*n* = 5 per group; OrientBio, Seongnam, Republic of Korea) received primary immunization, followed by booster immunization on day 21 using the same concentration of bovine type II collagen and incomplete Freund’s adjuvant (Chondrex). Intradermal injections were administered at the base of the tail. Arthritis severity was monitored for 28 days after the first injection, with scoring based on hind paw swelling and clinical assessment [19]. To evaluate therapeutic efficacy, 1 × 10^6^ AMM, AMM/I, or PBS were injected intraperitoneally twice a week (on days 0 and 7) once the arthritis score reached 3 or higher.

### 4.8. Flow Cytometry Analysis

Tregs and Treg cell populations were assessed using flow cytometry. The antibodies used were phycoerythrin (PE)-conjugated rat anti-mouse CD4 (1:400, eBioscience, San Diego, CA, USA, Cat #12-0041-82), fluorescein isothiocyanate (FITC)-conjugated rat anti-mouse IL-17A (1:300, eBioscience, Cat #11-7177-81), and FITC-conjugated rat anti-mouse CD25 (1:350, eBioscience, Cat #11-0251-82). Data analysis was performed using the CellQuest software v 5.1 (BD).

### 4.9. Histological Analysis

To collect cartilage samples, the mice were euthanized using CO_2_ gas and the tissues were dissected. The limbs and paws were fixed overnight in 4% paraformaldehyde and subsequently decalcified. The cartilage was then embedded in optimal cutting temperature (OCT) compound and cryosectioned at a thickness of 10 µm. Inflammation was assessed by staining sections with Hematoxylin and Eosin (H&E). Additionally, to confirm cartilage destruction in the CIA model, specimens were stained with safranin O/fast green (Science Cell) according to the manufacturer’s instructions. Cartilage degradation was evaluated using a scoring system ranging from 0 to 3, where 0 represented no loss of proteoglycans and 3 indicated complete loss of staining for proteoglycans [40]. Pathological changes were further scored based on the degree of inflammation in the cartilage and bone destruction using a previously established scale [41]: 0, normal synovium, 1 = synovial membrane hypertrophy and cell infiltrates, 2 = presence of pannus and cartilage erosion, 3 = significant erosion of the cartilage and subchondral bone; and 4, loss of joint integrity and ankylosis.

### 4.10. Statistical Analysis

All data are expressed as mean ± standard deviation (SD). Student’s *t*-test was used for comparisons between two groups, while analysis of variance (ANOVA) with Bonferroni’s multiple comparison test was used for comparisons involving multiple groups, using SPSS v13.0. Statistical significance was set at *p* < 0.05. significant [38].

## 5. Conclusions

In summary, the administration of AMM/I demonstrated significant anti-arthritogenic effects through multiple mechanisms, including the suppression of T-cell activation, inhibition of inflammatory responses, and promotion of Treg generation. Importantly, transplantation of AMM/I led to a notable amelioration of the condition in a mouse CIA model. These findings suggest that AMM/I represent potential means of therapeutic intervention for the treatment of arthritic joint tissues. This study, while providing valuable insights, possesses certain limitations. Firstly, the fate of transplanted cells remained unmonitored. Secondly, the utilization of a mouse model may not replicate human disease conditions. Thirdly, the autonomous or non-autonomous impacts of IGF-1 on cytokine production and its anti-inflammatory therapeutic mechanism, particularly in its interaction with other immune cells, necessitate further investigation. Future research endeavors should examine the in vivo tracking of AMM/I, the translation of current findings into human trials, and the assessment of the long-term safety and efficacy of AMM/I treatment for arthritis.

## Data Availability

The data presented in this study are available on request from the corresponding author upon reasonable request.

## Supplementary Materials

The following supporting information can be downloaded at: https://www.mdpi.com/article/10.3390/ijms25084442/s1.
