# Supplementary material for: IGF-1 Genome-Edited Human MSCs Exhibit Robust Anti-Arthritogenicity in Collagen-Induced Arthritis
Source: Int J Mol Sci. 2024 Apr 18;25(8):4442. doi: 10.3390/ijms25084442 (PMC11050354; doi:10.3390/ijms25084442)

Supplementary Data

Supplementary Figure Legend

Supplementary Figure S1. Characteristics of AMM/I. (A) Microscopic view of AMM/I (passage 4). Bar: 500μm. (B) Representative FACS surface markers of AMM/I exhibiting MSC-specific cell surface markers. Black colour represents the isotype control and red colour represents the specific monoclonal antibody.

Supplementary Figure S1.

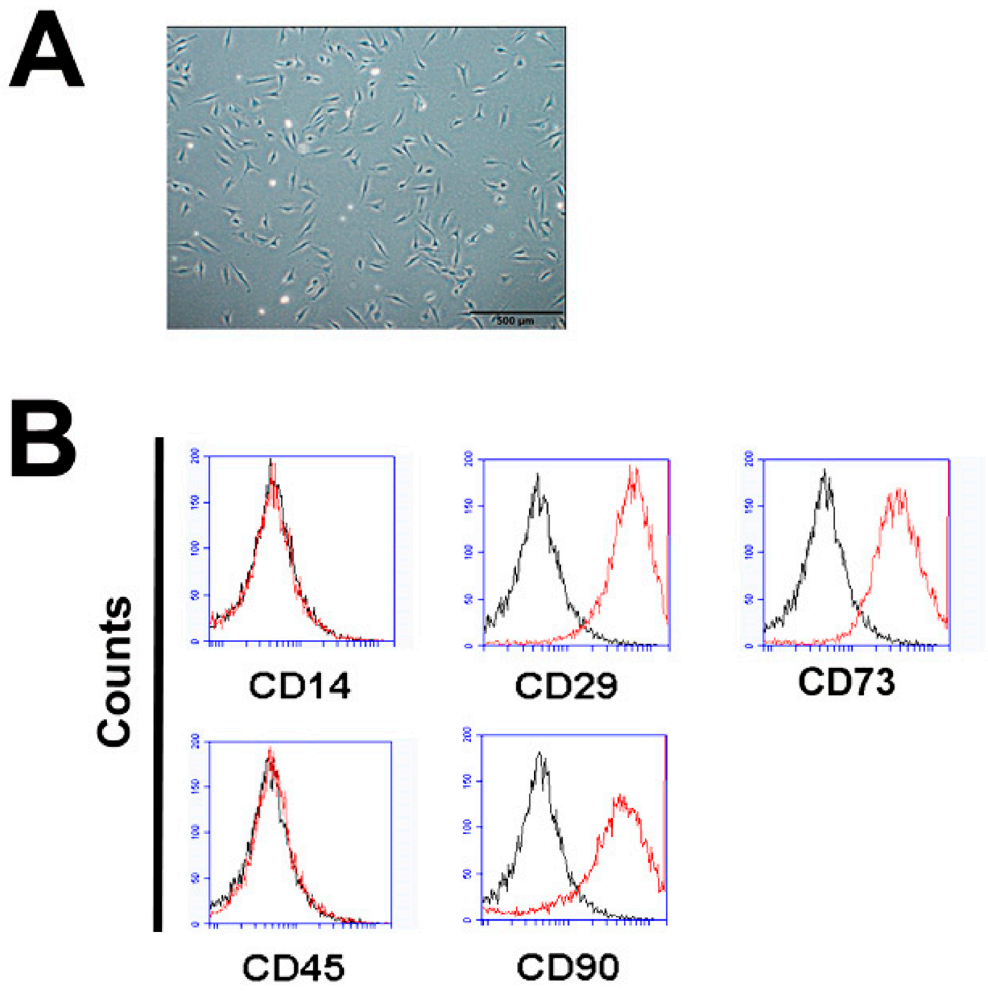

Supplement: Supplementary file 1 [file ijms-25-04442-s001.zip › ijms-2951892-supplementary.pdf]
